# A Prochlorperazine-Induced Decrease in Autonomous Muscle Activity during Hindlimb Unloading Is Accompanied by Preserved Slow Myosin mRNA Expression

**DOI:** 10.3390/cimb45070354

**Published:** 2023-06-30

**Authors:** Kristina A. Sharlo, Irina D. Lvova, Sergey A. Tyganov, Ksenia V. Sergeeva, Vitaly Y. Kalashnikov, Ekaterina P. Kalashnikova, Timur M. Mirzoev, Grigoriy R. Kalamkarov, Tatiana F. Shevchenko, Boris S. Shenkman

**Affiliations:** 1 Myology Laboratory, Institute of Biomedical Problems, Russian Academy of Sciences, 123007 Moscow, Russia; 2 Emanuel Institute of Biochemical Physics, Russian Academy of Sciences, 119334 Moscow, Russia

**Keywords:** hindlimb unloading, muscle activity, MyHC, MAP-kinase, soleus muscle

## Abstract

Skeletal muscle disuse leads to pathological muscle activity as well as to slow-to-fast fiber-type transformation. Fast-type fibers are more fatigable than slow-type, so this transformation leads to a decline in muscle function. Prochlorperazine injections previously were shown to attenuate autonomous rat soleus muscle electrical activity under unloading conditions. In this study, we found that prochlorperazine blocks slow-to-fast fiber-type transformation in disused skeletal muscles of rats, possibly through affecting calcium and ROS-related signaling.

## 1. Introduction

A significant downregulation mRNA expression of the slow-type isoform of myosin heavy chains (MyHCI (β)) and a concomitant upregulation of mRNA expression of the fast-type isoforms of myosin heavy chains (MyHC IId/x and MyHC IIB) are often observed in rodent soleus muscle during hindlimb suspension (HS) and are considered as consequences of disuse [1]. However, if HS lasts longer than 3 days, the soleus muscle cannot be considered to be in a state of complete rest. In 1987, Alford et al. showed that by the 3rd day of hindlimb suspension the values of integrated electromyographic (EMG) activity start rising gradually during HS and by the end of the second week of HS the EMG values are comparable to that of the vivarium control animals [2]. This phenomenon, which can be called “autonomous” neuromuscular activity, was reproduced under similar conditions by other authors [3,4]. This autonomous activity, contrary to expectations, does not pre-vent the development of atrophic changes in terms of mRNA expression patterns of the slow and fast isoforms of MyHC. The nature of this activity has remained unexplored until recently. At the same time, in another disuse model, a spinal cord injury (SCI), it was shown that shortly after injury an increase in electrical activity of skeletal muscles, in-cluding rodent soleus muscle, is observed subsequently leading to spasticity, which is a well-known consequence of spinal lesions in animals and humans. This SCI-induced muscle activity was shown to be a result of a significant decrease in the expression of a potassium-chloride co-transporter KCC-2 in lumbar motoneurons with a subsequent change in the direction of the chloride current and inversion of the resting membrane potential [5]. These changes lead to a sharp increase in the excitability of motoneurons that start generating impulses in response to inhibitory mediators [5]. A SCI-induced down-regulation of KCC2 can be prevented by treatment by prochlorperazine, an antipsychotic drug and an enhancer of KCC2 activity [6]. In our laboratory, a significant decrease in the protein content of KCC-2 was recently shown in lumbar motoneurons of rats subjected to 7-day HS [7]. Using implanted electrodes, we have registered the autonomous EMG activity of rat soleus muscle after 2 days of HS. Prochlorperazine treatment during one week of hindlimb suspension prevented a decrease in KCC2 protein expression and reduced the level of electrical activity of the soleus muscle [7]. These results allow us to further investigate the intramuscular consequences of the reduced autonomous activity of rat soleus muscle under unloading conditions. First of all, this concerns the effect of the autonomous activity on alterations in mRNA expression of the slow and fast isoforms of MyHC observed during mechanical unloading, as well as molecular regulators that ensure these changes. The study was aimed to analyze mRNA content of the slow and fast MyHC isoforms and molecular regulators of MyHC expression and markers of mitochondrial biogenesis during prochlorperazine administration that significantly reduces the tonic autonomous electrical activity of rat soleus muscle under conditions of simulated gravitational unloading.

## 2. Materials and Methods

### 2.1. Experimental Design

All animals were treated in accordance with EC Directive 86/609/EEC for animal experiments. All procedures with rats were approved by the Biomedicine Ethics Committee of the IBMP RAS (protocol no. 575, 4 December 2021).

Forty-eight Wistar rats (2.5-month-old, 196 ± 10 g) were divided into 3 groups: cage control (C), 7-day hindlimb suspension (7HS), and 7-day HS + intraperitoneal injections of prochlorperazine dimaleate salt (#P9178; Sigma-Aldrich, St. Louis, MO, USA), 5 mg/kg body weight (7HS + P). Prochlorperazine was injected twice a day in a saline containing 0.1% DMSO. Rats from the C and 7HS groups received equivalent volume of saline + DMSO without prochlorperazine. After the experiment, the animals were narcotized by means of an intraperitoneal injection of a tribromoethanol (750 mg/kg #T48402; Sigma-Aldrich, St. Louis, MO, USA), and sacrificed via neck dislocation. Changes in soleus weight, body weight, and soleus weight/body weight ratio are shown in Table 1.

Half of the experimental animals were injected with substances required for NO detection (as described below), while the soleus muscles of another half of the rats were frozen in liquid nitrogen until further biochemical assays.

Mechanical unloading was carried out using the standard model of rat hindlimb suspension (HS) as described previously [8]. Briefly, a U-shaped wire, fixed on the rat’s tail with a strip of adhesive tape, was attached via a stainless-steel chain to a swivel that freely moves along a metal bar on the top of the cage. In this model, rats are able to freely move around the cage using their forelimbs but their hindlimbs do not reach the floor or the cage walls.

### 2.2. NO Content Detection

Nitric oxide (NO) levels in skeletal muscles were determined as described in our previous paper [9]. Relative NO content in muscle tissue was assessed using electron paramagnetic resonance (EPR) spectroscopy. The rats were injected with diethyldithiocarbamate (DETC) (500 mg/kg) that is used as a spin trap for NO-forming paramagnetic complexes and has a characteristic EPR spectrum. Then, the rats received an intramuscular injection of an aqueous solution containing 29 mM FeSO4 and 116 mM sodium citrate (2 mL/kg body mass). Thirty minutes later, the rats were euthanized, and the muscles were quickly frozen in LN_2_. A Bruker EMX-8 EPR spectrometer (Billerica, Massachusetts) was used to register EPR signals.

### 2.3. ATP Content Detection

ATP content in skeletal muscles was assessed using an ATP Colorimetric/Fluorometric Assay Kit (MAK190; Sigma, St. Louis, MO, USA) according to the manufacturer’s instructions as previously described [10].

### 2.4. Detection of Superoxide Anion

Frozen skeletal muscle sections (8 μm) were obtained with the help of a Leica CM 1900 cryostat (Braunschweig, Germany). The obtained sections were dried at room temperature for 15 min and incubated in PBST for 15 min. Dihydroethidium (DHE), a well-known ROS indicator, was diluted in PBS to a final concentration of 2 µmol/L. After that, 200 µL of DHE–PBS solution was applied to tissue sections and incubated at 37 °C in dark for 30 min. Images were captured using a fluorescence microscope: Leica Q500MC (Wetzlar, Germany) (at 20× objective magnification). Excitation and emission wavelengths were 520 nm and 610 nm, respectively.

### 2.5. Total Protein Fraction Preparation

Details concerning methods of protein extraction and the determination of protein concentration can be found in our previous paper [11].

### 2.6. Western Blot Analysis

Gel-electrophoresis and immunoblotting were performed as previously described [11]. In brief, protein samples were separated using 10% SDS-PAGE and then transferred to nitrocellulose membranes (Bio-Rad Laboratories, Hercules, CA, USA) in Western Blot Transfer Buffer (25 mM Tris, 192 mM glycine, pH 8.3, 20% ethanol, 0.04% SDS) at 100 V and 4 °C in the mini-Trans-Blot system (Bio-Rad Laboratories, Hercules, CA, USA) for 120 min. The membranes were incubated with the following primary antibodies: total GSK3β and phospho-(Ser 9) GSK3β (Cell Signaling Technology, Inc., Danvers, MA, USA, 1:1000 # 9322, # 12456), total p38 MAPK (1:500, Cell Signaling Technology, Inc., Danvers, MA, USA, #9212), phospho-(Tyr180/Thr182) p38 MAPK (1:500, GeneTex, Inc., Irvine, CA, USA, # GTX59567), total JNK (1:1000, SAB4200176, Sigma-Aldrich, Burlington, MA, USA) and phospho-(Thr183/Tyr185) JNK (1:1000, 44-682G, Thermo Fisher Scientific, Waltham, MA, USA;), total glycogen synthase 1 and phospho-(Ser 641) glycogen synthase 1 (Abcam, Waltham, MA, USA, 1:10,000, #ab40810, #ab8123), phospho-(Ser 79) ACC (1:1000, Cell Signaling Technology, Inc., Danvers, MA, USA, #3661) and total ACC (1:2000, Cell Signaling Technology, Inc., Danvers, MA, USA, #2072), total CaMKII (CSB-PA061493, Cusabio, Wuhan, China 1:1000) and phospho-(Thr180/Tyr-182) CaMKII (CSB-PA283993, Cusabio, Wuhan, China, 1:1000), GAPDH (1:10,000, G 041, ABM, Richmond, BC, Canada). Secondary antibodies: horseradish peroxidase-conjugated goat anti-rabbit (1:30,000, #111-035-003, Jackson Immuno Research, West Grove, PA, USA) or goat anti-mouse (1:20,000, #1706516, Bio-Rad, Hercules, CA, USA). The protein expression signals were detected using Clarity Western ECL Substrate (Bio-Rad Laboratories, Hercules, CA, USA) and a C-DiGit Blot Scanner (LI-COR Biotechnology, Lincoln, NE, USA).

### 2.7. RNA Extraction, Reverse Transcription and PCR

RNA extraction and RT-PCR analysis were performed as reported previously [11]. Primers are listed in Table 2. The obtained data were quantified using the Pfaffl method (32). RPL19 was chosen as a reference gene since its expression did not significantly differ across the groups.

### 2.8. MyHCs Immunostaining

A detailed description for immunostaining-based assessment of fiber cross-sectional area (CSA) and the percentage of slow and fast muscle fibers can be found in our previous reports [12].

### 2.9. NFATc1 Detection in Muscle Nuclei

A method of NFATc1 detection in muscle cryosections has been described in our previous paper [12]. The following primary antibodies were used: antibodies against NFATc1 (1:500, BD Biosciences, Franklin Lakes, NJ, USA) and antibodies against pericentriolar material 1 (PCM1) (1: 1000, HPA023370, Sigma, Burlington, MA, USA) to label myonuclei [13]. Alexa Fluor 488 nm and Alexa Fluor 546 nm dye-conjugated antibodies served as secondary antibodies (dilution range 1:500–1:2000, Molecular Probes, Eugine, OR, USA).

### 2.10. Statistical Analysis

Most data are shown as median and interquartile range (0.25–0.75) ± the minimum and the maximum. Body weight, muscle weight, and immunohistochemical data are shown as M ± SEM. Differences between the groups were analyzed using the Kruskal–Wallis test followed by Dunn’s test or using ANOVA followed by the Bonferroni test (for immunohistochemical data). A *p* value less than 0.05 was regarded as statistically significant.

## 3. Results

Muscle activity usually leads to NO and ROS accumulation [14,15], a decrease in ATP (resulting in the activation of the key muscle energy sensor AMP-activated protein kinase (AMPK)), and activation of calcium-dependent signaling [16]. Prochlorperazine was shown to affect muscle EMG activity; therefore, we evaluated the state of these key markers of muscle activity in the present study.

**Figure 1:**
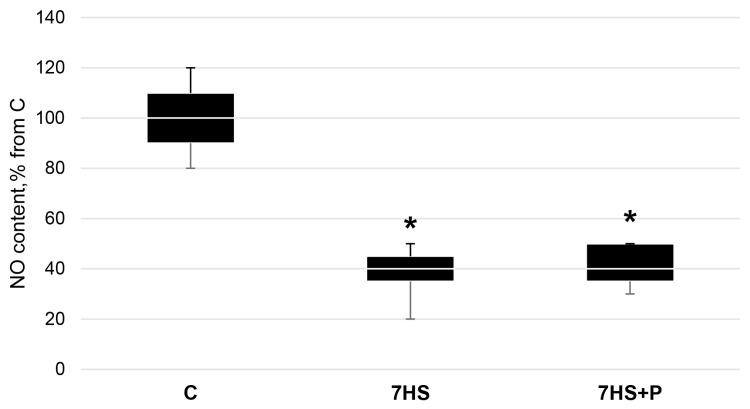
NO content in the control group (C), 7-day hindlimb-suspended group (7HS), 7-day hindlimb suspended group with prochlorperazine (7HS + P), Data shown as % of the control group. *—significant difference from the control group (*p* < 0.05). Box plots show 25–75 percentiles and median values and the whiskers represent the minimum and the maximum; *n* = 8/group.

In the 7HS group, the content of NO in the soleus muscle was 60% less (*p* < 0.05) than that in the control group and did not differ from the 7HS + P group. The NO levels in both the 7HS and 7HS + P groups significantly decreased (*p* < 0.05) compared to the C group (Figure 1).

**Figure 2:**
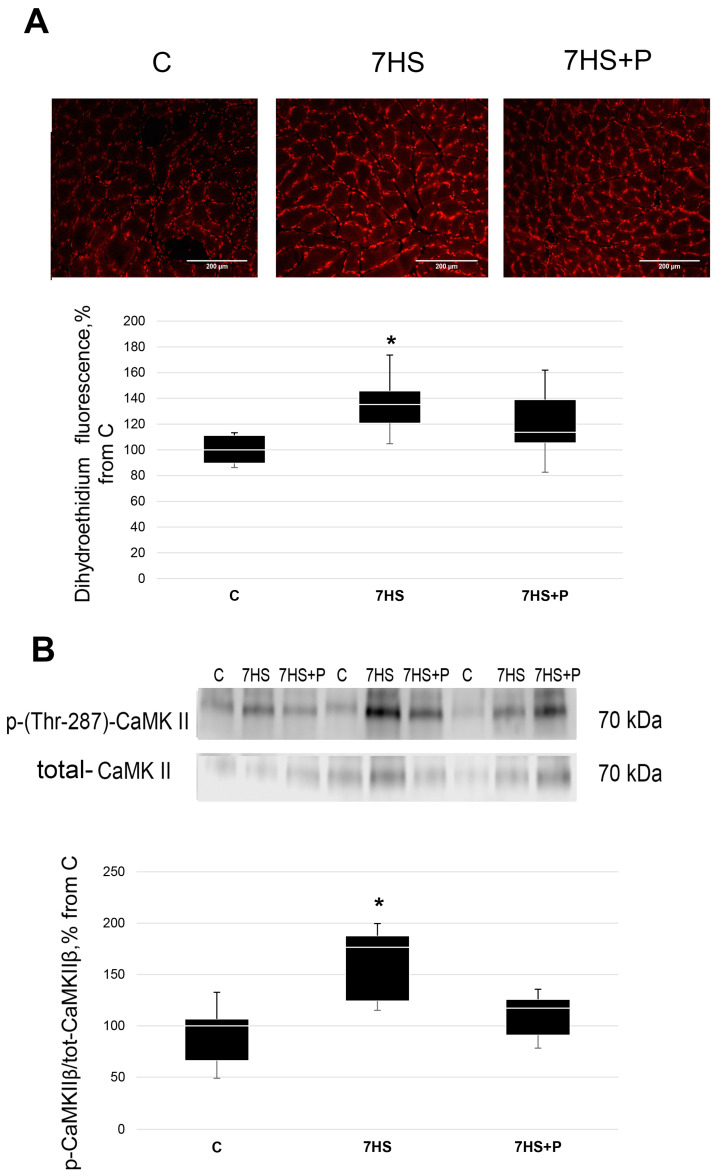
The content of superoxide anions measured via dihydroethidium fluorescence signal (**A**) and phospho-(Thr-287)-CaMK II content (**B**) in the control group (C), 7-day hindlimb-suspended group (7HS), and 7-day hindlimb suspended group with prochlorperazine (7HS + P). Data shown as % of the control group. *—significant difference from the control group (*p* < 0.05). Box plots show 25–75 percentiles and median values, and the whiskers represent the minimum and the maximum; *n* = 8/group.

The content of superoxide anions measured using the dihydroethidium fluorescence signal was 35% greater (*p* < 0.05) in the unloading group vs. the control rats. In the 7HS + P group, the dihydroethidium fluorescence signal was only 13% above the control group and did not significantly differ (*p* > 0.05) from either the 7HS or the C group (Figure 2A). In the 7HS group, the content of phospho-(Thr-287)-CaMK II significantly increased by 70% (*p* < 0.05) compared to the C group (Figure 2B). In the 7HS + P group, CaMK II phosphorylation did not differ from the control group (Figure 2B).

**Figure 3:**
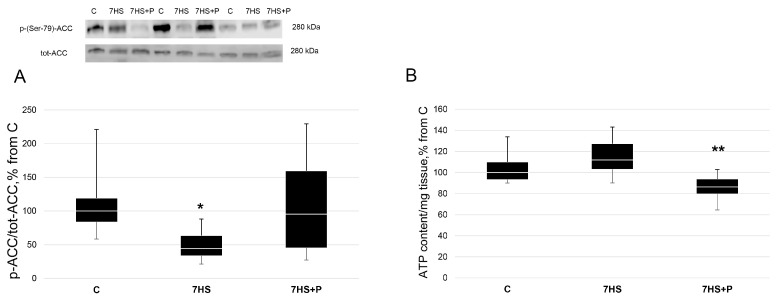
Phosphorylation status of ACC (Ser 79) phosphorylation (**A**) and ATP content/mg tissue (**B**) in the control group (C), 7-day hindlimb-suspended group (7HS), and 7-day hindlimb suspended group with prochlorperazine (7HS + P). Data shown as % of the control group. *—significant difference from the control group. **—significant difference from the 7HS group (*p* < 0.05). Box plots show 25–75 percentiles and median values, and the whiskers represent the minimum and the maximum; *n* = 8/group.

ACC (Ser 79) phosphorylation (a downstream target of AMPK) in the rat soleus muscle was twofold less (*p* < 0.05) in the 7HS group vs. the C group, while in the 7HS + P group ACC (Ser 79) phosphorylation did not differ from the C group (Figure 3A). The concentration of ATP did not differ between the C and 7HS groups; however, it was significantly less (*p* < 0.05) in the 7HS + P group vs. the 7HS group (Figure 3B).

The percentage of slow and fast fibers in skeletal muscle is strictly regulated by the activation pattern of motoneurons controlling muscle [17]. The proportion of slow-twitch and fast-twitch fibers is determined by the expression of different myosin genes; therefore, we evaluated the mRNA expression levels of slow and fast isoforms of myosin heavy chains (MyHC).

**Figure 4:**
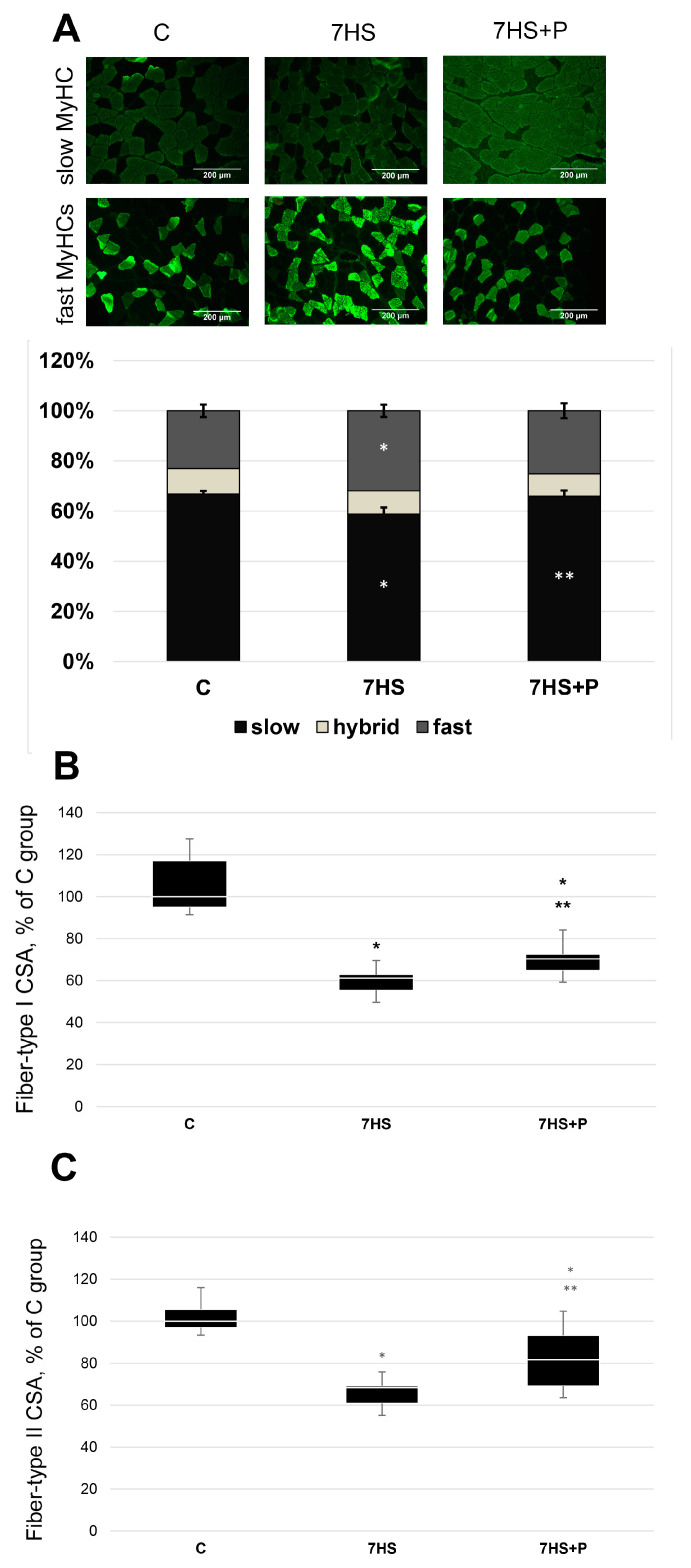
Immunohistochemical analysis of the percentage of slow-type and fast-type muscle fibers (**A**), cross-sectional area of type I (**B**) and type II (**C**) muscle fibers in rat soleus muscle in the control group (C), 7-day hindlimb-suspended group (7HS), and 7-day hindlimb suspended group with prochlorperazine (7HS + P). Data are M ± SEM expressed as % of the control group. *—significant difference from the control group. **—significant difference from the 7HS group (*p* < 0.05). The data are represented as mean value ± SEM. *n* = 8/group.

In the C group, the percentage of slow-twitch fibers in the soleus muscle was significantly greater (*p* < 0.05) than in the 7HS group (Figure 4). In the 7HS + P group, the percentage of slow-type fibers was 65%, which was significantly higher (*p* < 0.05) than in the 7HS group and did not differ from the C group (Figure 4). In the C group, the percentage of fast-twitch fibers in the soleus was 22%, which was significantly less (*p* < 0.05) than in the soleus muscle of the unloaded animals (34% of fast-type fibers) (Figure 4). In the unloaded rats treated with prochlorperazine, the percentage of fast-twitch fibers was 25% and did not differ from the C group (Figure 4). The cross-sectional area (CSA) of slow-type and fast-type myofibers decreased (*p* < 0.05) in both the 7HS group vs. C group (by 39 and 28%, respectively), while in the 7HS + P group, fiber CSA was significantly greater (*p* < 0.05) than in the 7HS group, but significantly less (*p* < 0.05) than in the C group (Figure 4B,C).

**Figure 5:**
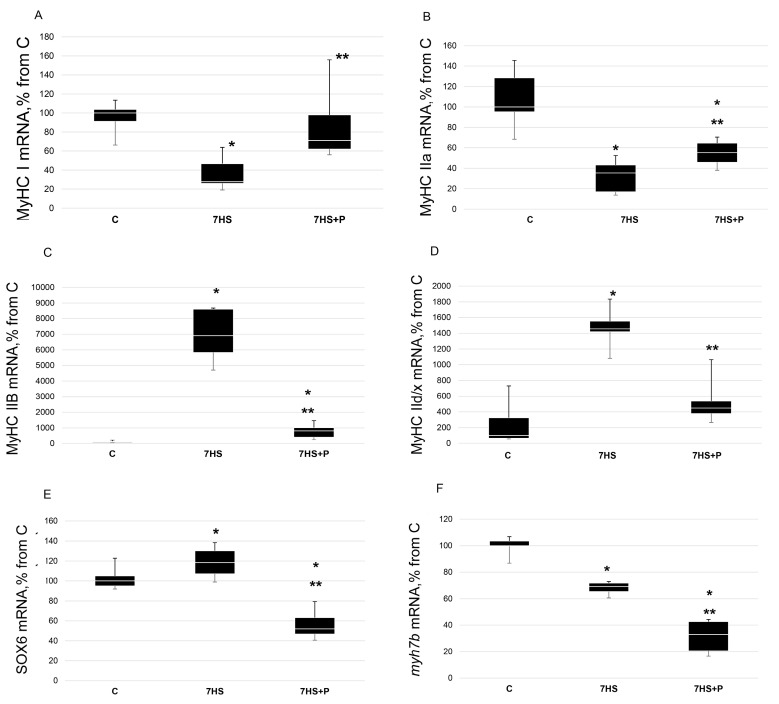
RT-PCR analysis of MyHC I(β) (**A**), MyHC IIa (**B**), MyHC IIB (**C**), MyHC IId/x (**D**), SOX6 (**E**), and myh7b (**F**) mRNA content in rat soleus muscle in the control group (C), 7-day hindlimb-suspended group (7HS), and 7-day hindlimb suspended group with prochlorperazine (7HS + P). Data shown as % of the control group. *—significant difference from the control group. **—significant difference from 7HS group (*p* < 0.05). Box plots show 25–75 percentiles and median values, and the whiskers represent the minimum and the maximum; *n* = 8/group.

In the unloaded rats, the content of slow-type myosin mRNA was 73% lower (*p* < 0.05) than that in the control group, while in the 7HS + P group, it was significantly greater (*p* < 0.05) than in the unloaded animals (only 34% lower than in the C group) and did not significantly differ from the control rats (Figure 5A). The content of myosin IIa mRNA was lower (*p* < 0.05) in both the unloaded rats and unloaded rats treated with prochlorperazine compared to the C group (by 61% and 44%, respectively); however, MyHC IIa mRNA expression was greater (*p* < 0.05) in the 7HS + P group than in the 7HS group (Figure 5B). In the 7HS group, the content of myosin IIB mRNA was 60-fold greater (*p* < 0.05) vs. the C group, while it was only 8-fold greater (*p* < 0.05) in the 7HS + P group (*p* < 0.05). In the 7HS + P group, the content of myosin IIB mRNA was significantly lower (*p* < 0.05) vs. the 7HS group (Figure 5C). In the unloaded rats, the content of IId/x myosin isoform was 14.7-fold higher (*p* < 0.05) compared to the C group, and in the 7HS + P group, MyHC IId/x expression was 4.5-fold higher relative to the control rats (not statistically significant vs. the C group) (Figure 5D).

Slow-type myosin mRNA expression can activate the expression of the myh7b gene, the product of which, mir-499, blocks the expression of SOX6, a repressor of slow-type MyHC genes [18]

The level of mRNA expression of SOX6 was 20% higher (*p* < 0.05) in the unloaded rats vs. the control animals. In the 7HS + P group, the content of SOX6 mRNA was lower (*p* < 0.05) than in both the C group and 7HS groups. Myosin 7b mRNA content was 30% less (*p* < 0.05) in the 7HS group vs. the C group and 70% less (*p* < 0.05) in 7HS + P group vs. the C group. The content of myosin 7b mRNA in the 7HS + P group was significantly less (*p* < 0.05) than in the 7HS group (Figure 5E,F).

Transcription factor NFATc1 is a nerve activity sensor, involved in the regulation of slow-type myosin gene expression in response to a slow-type mode of myofiber activity [17]; therefore, NFATc1 myonuclear content and the content of the marker of its activity, MCIP1.4, was analyzed in our study.

**Figure 6:**
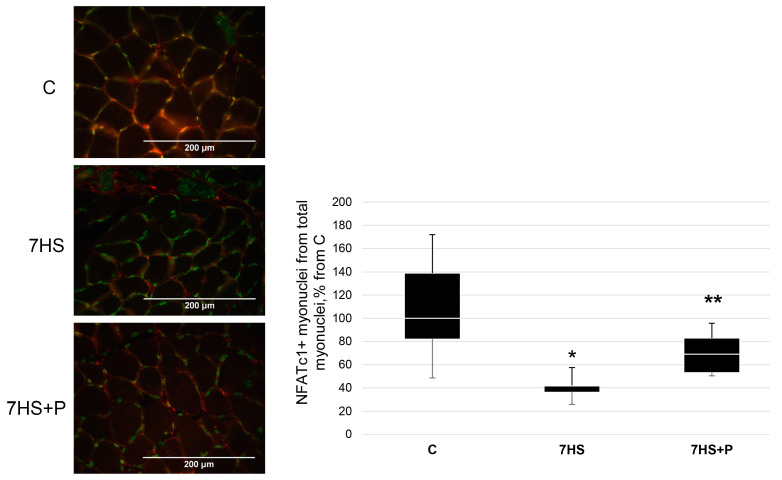
Immunohistochemical analysis of the percentage of NFATc1-positive myonuclei in the control group (C), 7-day hindlimb-suspended group (7HS), and 7-day hindlimb suspended group with prochlorperazine (7HS + P). NFATc1 stained red; myonuclei are labelled with PCM1 (green). Data shown as % of the control group. *—significant difference from the control group. **—significant difference from the 7HS group (*p* < 0.05). Box plots show 25–75 percentiles and median values, and the whiskers represent the minimum and the maximum; *n* = 8/group.

The percentage of NFATc1-positive nuclei in the 7HS group was 60% less (*p* < 0.05) than in the C group (Figure 6). In the 7HS + P group, the number of NFATc1-positive nuclei was only 25% less (*p* < 0.05) than in the C group, so that the values in the 7HS + P did not significantly differ from the C group (Figure 6).

**Figure 7:**
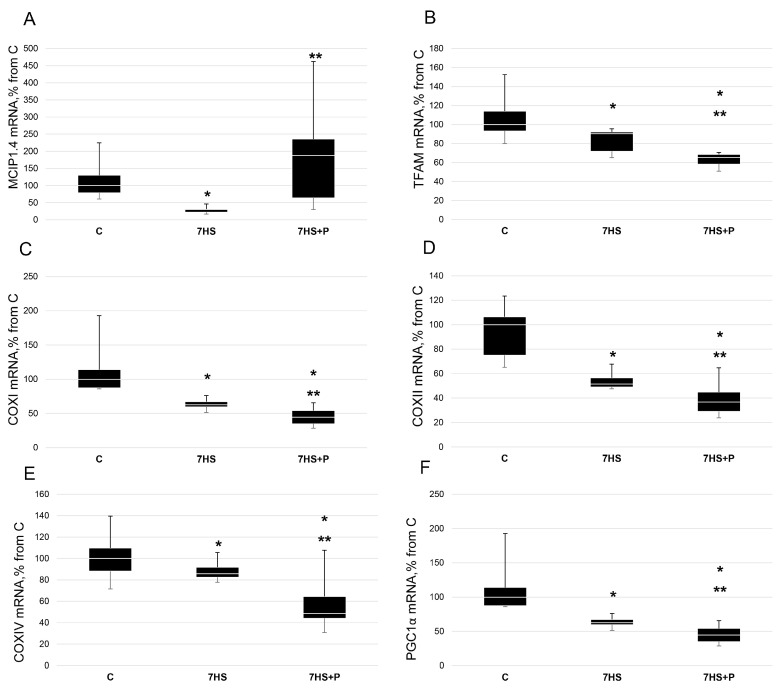
RT-PCR analysis of MCIP1.4 (**A**), TFAM (**B**), COXI (**C**), COXII (**D**), COXIV (**E**), and PGC1α (**F**) mRNA content in rat soleus muscle in the control group (C), 7-day hindlimb-suspended group (7HS), and 7-day hindlimb suspended group with prochlorperazine (7HS + P). Data shown as % of the control group. *—significant difference from the control group. **—significant difference from the 7HS group (*p* < 0.05). Box plots show 25–75 percentiles and median values, and the whiskers represent the minimum and the maximum; *n* = 8/group.

MCIP1.4 mRNA content was 60% lower (*p* < 0.05) in the 7HS group vs. the C group (*p* < 0.05) (Figure 7A). In the 7HS + P group, MCIP1.4 mRNA expression was 87% greater (*p* < 0.05) than in the C group (Figure 7A).

In skeletal muscle, slow fiber type formation often includes the activation of genes that control oxidative metabolism, including mitochondrial function. For example, the key regulator of mitochondrial biogenesis, PGC1α, can activate slow-type myosin expression, while slow-type myosin gene expression can lead to PGC1α upregulation via the myh7b/FNIP/AMPK signaling pathway [19]. In the 7HS group, the expression levels of TFAM, COXI COXII, COXIV, and PGC1α were significantly lower (*p* < 0.05) than those in the C group (by 10%, 38%, 50%, 20%, and 33%, respectively) (Figure 7B–F). In the 7HS + P group, the mRNA expression levels of TFAM, COXI COXII, COXIV, and PGC1α were significantly lower (*p* < 0.05) than in both the C and 7HS groups (Figure 7B–F).

**Figure 8:**
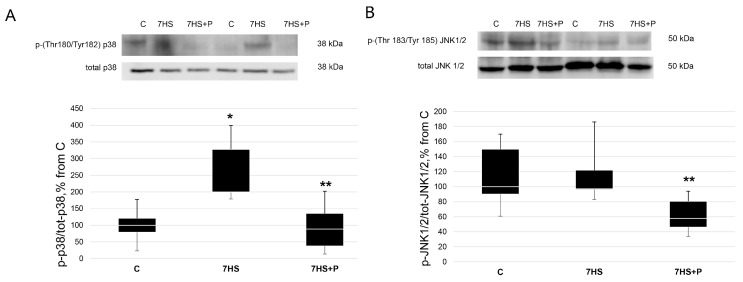
Western blot of p-(Thr180/Tyr-182)-p38 (**A**) and p-(Thr183/Tyr185)-JNK1/2 (**B**) content in rat soleus muscle in the control group (C), 7-day hindlimb-suspended group (7HS), and 7-day hindlimb suspended group with prochlorperazine (7HS + P). Data shown as % of the control group. *—significant difference from the control group. **—significant difference from 7HS group. Box plots show 25–75 percentiles and median values, and the whiskers represent the minimum and the maximum; *n* = 8/group.

NFATc1 can be phosphorylated and exported from the myonuclei by three protein kinases, p38, JNK, and GSK3beta; therefore, we evaluated the phosphorylation levels of these kinases in order to detect which one of these kinases could be involved in the observed changes in NFATc1 myonuclear content in the 7HS + P group. The content of p-(Thr180/Tyr182)-p38 MAP kinase in the 7HS group was significantly greater (+150%, *p* < 0.05) than in the control rats (Figure 8A). In the 7HS + P group, p38 MAP kinase phosphorylation decreased (*p* < 0.05) compared to the 7HS group and did not differ from the control values (Figure 8A). The content of p-(Thr183/Tyr185) JNK1/2 did not differ between the C and 7HS groups; however, in the 7HS + P group, JNK1/2 phosphorylation significantly decreased (*p* < 0.05) relative to both the C and 7HS groups (Figure 8B).

**Figure 9:**
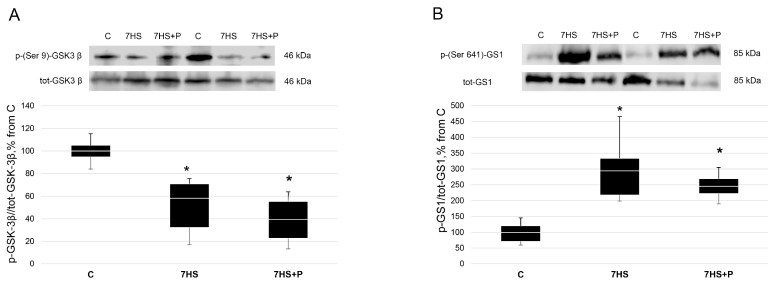
Western blot of p-(Ser-9)-GSK-3β (**A**) and p-(Ser-641)-GS1 (**B**) content in rat soleus muscle in the control group (C), 7-day hindlimb-suspended group (7HS), and 7-day hindlimb suspended group with prochlorperazine (7HS + P). Data shown as % of the control group. *—significant difference from the control group. Box plots show 25–75 percentiles and median values, and the whiskers represent the minimum and the maximum; *n* = 8/group.

The content of p-(Ser-9)-GSK-3β was significantly lower (*p* < 0.05) in both the 7HS and 7HS + P groups vs. the C group (Figure 9A). The content of GSK-3β target p-(Ser-641)-GS1 (a downstream target of GSK-3β) was about 1.5-fold greater (*p* < 0.05) in both the 7HS and 7HS + P groups compared to the C group (Figure 9B).

## 4. Discussion

Previous studies clearly demonstrated that there is a sharp decline in rat soleus EMG activity at the early stage of HS (1–2 days) [2,4,7]. However, soleus muscle electrical activity starts increasing after 48 h of exposure to HS, and after 3 days of HS, soleus muscle EMG activity significantly differs from the values recorded immediately after the onset of HS [7]. Furthermore, it has been shown that rat soleus muscle EMG activity progressively increases from *day 2* to *day 6* of HS [7]. We hypothesized that this rise in soleus muscle EMG activity could be associated with the loss of KCC-2 in lumbar motoneurons that was observed in our previous study following 7-day [7]. Indeed, daily prochlorperazine administration over the course of 7-day HS not only prevented an HS-induced decrease in KCC2 protein content but also attenuated autonomous soleus muscle [7]. The purpose of the present study was to elucidate the signaling consequences of a decrease in autonomous soleus muscle activity under mechanical unloading, i.e., conditions that are close to complete disuse.

Our initial hypothesis suggested that with prochlorperazine treatment (that is able to reduce autonomous soleus muscle activity), the main disuse-induced signaling effects would not change or would be more pronounced compared to the vehicle-treated HS animals. Indeed, a number of indicators of mitochondrial signaling and the mRNA expression of a main regulator of mitochondrial biogenesis, PGC1α, showed a deeper decrease in the HS prochlorperazine-treated rats compared to the rats from the 7HS group. This fact indicates that mitochondrial signaling is directly dependent on soleus muscle activity. However, to date, muscle-activity-dependent messengers that underlie such dependence are not known. One of the important regulators of intracellular signaling is GSK3β (that can be regulated by both Akt and NO/cGMP-dependent signaling (for rev. see [20]), which demonstrated a similar degree of inhibitory Ser 9 phosphorylation in the soleus muscle of rats from both the HS and HS + P groups. It has been shown that activated GSK3β can contribute to a decrease in PGC1α expression [21]. However, we did not find a decrease in GSK3β (Ser 9) phosphorylation (indicative of GSK3β activation) in the HS rats treated with prochlorperazine compared to the 7HS rats. Hence, based on the data obtained in the present study, it should be pointed out that changes in the expression of PGC1α in the 7HS + P group are apparently determined by some other factors. Thus, the results of the analysis of the effect of prochlorperazine on the markers of mitochondrial signaling allow us to put forward a plausible hypothesis that autonomous soleus muscle activity may play a compensatory role for this signaling pathway under conditions of mechanical unloading.

In most cases, mechanical unloading is accompanied by a change in the ratio of slow-type to fast-type muscle fibers caused by the transformation of a part of the slow fibers into the fast ones (for review see [22]). This fiber type shifting occurs due to a decrease in the expression of the slow MyHC isoform and an increase in the expression of the fast isoforms of MyHC [22]. As expected, in the present study, we observed a significant decline in the percentage of slow-type fibers and concomitant increase in the proportion of fast-type fibers in the 7HS group compared to the weight-bearing control rats. In addition, there was a significant decrease in the mRNA expression of MyHC I (β) and MyHC IIa and an increase in the mRNA expression of MyHC IId/x and MyHC IIB. Usually, such fiber type changes are explained by a decrease in muscle activity [2]. However, the proportion of the slow-type fibers in the soleus of HS rats treated with prochlorperazine (and thereby having lower soleus muscle electrical activity compared to the 7HS group) did not differ from the control values. Furthermore, the levels of MyHC I(β) and MyHC IIa mRNA expression in the 7HS + P rats were significantly higher than those in the 7HS rats. At the same time, the expression levels of the fast isoforms of MyHC were much lower in the HS rats treated with prochlorperazine compared to the 7HS group. Thus, a greater decrease in the soleus muscle activity in the 7HS + P group compared to the 7HS group paradoxically resulted in the maintenance of the expression patterns of the MyHC isoforms at levels that are close to the control conditions. Similar effects on the mRNA expression of the MyHC isoforms during HS were previously observed under the following conditions:

- an activation of AMP-activated protein kinase (AMPK) by AICAR or β-guanidinopropionic acid (mainly at the early stages of unloading) [23,24,25];
- an increase in the levels of nitric oxide (NO), for example, through the use of L-arginine, which contributes to NFATc1 dephosphorylation and its import to the myonuclei [9];
- an inhibition of protein kinases that phosphorylate NFATc1, promoting its export from the myonuclei (for example, p-38 MAPK or GSK3β) [11,26];
- an inhibition of histone deacetylase 4 (HDAC-4) [27].

In the present study, prochlorperazine administration during 7-day HS resulted in a significant decrease in ATP content in rat soleus muscle compared to rats from the 7HS group. There were no significant differences in the ATP content in the soleus muscles of rats from the 7HS group compared to the C group. Our laboratory has previously found an increase in ATP content in rat soleus muscles after 3 days of HS [10]. This result can be explained by the almost complete cessation of soleus muscle electrical activity during the first 3 days of unloading [4]. Interestingly, Gupta et al. (1989) have showed that ATP content in soleus muscle does not differ from baseline values after 7-day HS but is significantly reduced after 14-day HS relative to control animals [28]. Alterations in ATP content can be caused by both (1) changes in ATP utilization along with changes in soleus muscle contractile activity during the course of HS and (2) a decrease in ATP production due to an impaired mitochondrial apparatus. In the HS rats treated with prochlorperazine, soleus muscle activity was reduced compared to the group of vehicle-treated HS rats. Other things being equal, one would expect an accumulation of ATP content in the soleus muscle. However, the ATP content in the soleus muscle of the prochlorperazine-treated HS rats significantly decreased. The reason for this reduction in the ATP content could be associated with changes in the mitochondrial apparatus as a consequence of a downregulation in the expression of mitochondrial biogenesis regulators (see above). It is possible that due to a decrease in the ATP content in the prochlorperazine-treated rats (and despite the reduced soleus muscle activity in the 7HS + P group), the level of AMPK phosphorylation could increase. This is evidenced by an increased content of the phosphorylated form of ACC, a marker of AMPK activity, in the 7HS + P group compared to the 7HS group. This increase in the AMPK activity could be one of the reasons for the absence of a reduction in MyHC I mRNA expression in the HS prochlorperazine-treated rats due to blocking the nuclear import of histone deacetylases, as it has been previously demonstrated in AICAR (AMPK activator)-treated rats during 24 h HS [29]. The analysis of the co-localization of myonuclei with NFATc1 showed that the number of NFATc1-labeled nuclei after 7-day HS was significantly less than in the control group, and prochlorperazine administration during HS attenuated the HS-induced decrease in the number of NFATc1-labeled nuclei. Obviously, in prochlorperazine-treated rats, the activity of one or more enzymes/pathways capable of NFATc1 phosphorylation (and thereby blocking NFATc1 import into myonuclei) could be reduced. According to the obtained resulted, the NO/cGMP/GSK3β pathway, which is known to regulate NFAT-dependent transcription [30], was not involved in the maintenance of the number of NFATc1-labeled nuclei in the 7HS + P group as prochlorperazine administration during HS did not affect the reduced levels of NO content and GSK3β (Ser9) phosphorylation in the soleus muscle. Our data suggest that two MAP kinases, p38 and JNK1/2, which are known to phosphorylate NFATc1 and promote its exclusion from the nucleus [26,31,32], could contribute to the maintenance of the number of NFATc1-labeled nuclei under HS and prochlorperazine administration since phosphorylation of both p38 and JNK1/2 was significantly reduced in the 7HS + P group compared to the 7HS group. It is known that accumulation of reactive oxygen species (ROS) may serve as a stimulus for the phosphorylation of MAP kinases [33]. Other upstream regulators of both JNK and p-38 MAP-kinases are calcium ions and CaMK II [34,35]. It is known that during rat hindlimb unloading, excessive calcium is accumulated in soleus myoplasm [36] accompanied by increased mitochondria-derived ROS [37]. In our study, we detected ROS and phospho-(Thr-287)-CaMK II accumulation in the 7HS group, which was partially prevented in the prochlorperazine-treated group. As CaMK II (Thr-287) phosphorylation depends on myoplasmic calcium levels [38], CaMK II (Thr-287) phosphorylation levels could be predictive of cytosolic calcium levels in the experimental groups.

If we assume that the autonomous tonic activity of the soleus muscle in the 7HS group contributes to the generation of free radicals and calcium ion accumulation leading to CaMK II phosphorylation, then almost complete elimination of this activity with prochlorperazine treatment can prevent the accumulation of Ca^2+^ and ROS, inactivate p38 and JNK, and counteract the decrease in the percentage of NFATc1-positive myonuclei. In this study, we also detected a partial prevention of both slow-type and fast-type myofiber atrophy. ROS accumulation as well as an excessive calcium increase can contribute to unloading-induced myofiber atrophy, so the mitigation of atrophy in the 7HS + P group may occur due to ROS and/or calcium-dependent mechanism inactivation. The prevention of the decrease in the nuclear content of NFATc1 and its transcriptional activity (as assessed according to MCIP1.4 expression) is one of the reasons for the prevention of reductions in the MyHC I(β) and MyHC IIa mRNA expression levels in the rat soleus muscle with prochlorperazine administration. The observed decline in ROS content in the 7HS + P group vs. the 7HS group could also explain the downregulation of PGC1alpha mRNA expression as well as PGC1alpha downstream regulators. PGC1alpha as well as other regulators of mitochondrial biogenesis are known to be activated by ROS production [39]. That is why the prochlorperazine-induced prevention of ROS accumulation could result in an exacerbation of the unloading-induced decline in PGC1alpha mRNA expression. Some of the signaling mechanisms that control slow myosin expression and the expression of oxidative metabolism genes are the same (such as AMPK or GSK3). However, the relative contribution of each of these mechanisms to slow myosin gene or oxidative metabolism gene expression could not be the same. In our study, in the 7HS + P group, we detected myonuclear accumulation of NFATc1, which is an activator of slow-type myosin expression but does not activate oxidative metabolism genes. This finding can explain the discrepancy between slow-type myosin and oxidative metabolism gene expression.

Administration of prochlorperazine to the HS rats also prevented an increase in the mRNA expression of both MyHC IId/x and MyHC IIB in the soleus muscle. Currently, there are few data in the literature on the activity-dependent regulation of the expression of the fast isoforms of MyHC. However, it appears that there are reciprocal relations in the expression of IIa and IId/x isoforms as a result of antisense transcription of their common promoter [40]. Therefore, the maintenance of a normal level of MyHC IIa mRNA expression could prevent an increase in MyHC IId/x mRNA expression.

It is worth noting that the experimental design used in the present study does not rule out the possibility of a direct effect of prochlorperazine on the soleus muscle fibers. However, there are no relevant data in the literature on the direct effects of prochlorperazine on intramuscular signaling. Therefore, it is difficult to discuss the results of the present study from such a perspective.

Thus, the discussion of the obtained results was mainly focused on a well-known effect of prochlorperazine associated with the attenuation of the excessive excitability of motor neurons with a decrease in neuromuscular activity and subsequent development of autonomous contractile activity with symptoms of spasticity [6].

## 5. Conclusions

Our study demonstrated that a significant decrease in the autonomous activity of unloaded rat soleus muscle with prochlorperazine treatment leads to a prevention of ROS accumulation and increased CaMK II phosphorylation accompanied by a deeper reduction in the expression levels of the regulators of mitochondrial biogenesis, inactivation of p38 and JNK1/2, and the maintenance of normal expression levels of slow and fast isoforms of MyHC.

## Figures and Tables

**Table 1:** Body weight, soleus muscle weight, and soleus weight/body weight ratio of the experimental animals. Data are shown as mean ± SEM. *—significant differences from the C group.

Group	Body Weight, g	Soleus Weight, mg	Soleus Weight/Body Weight, mg/g
C	191.4 ± 2.4	85.4 ± 3.2	44.6 ± 1.5
7HS	206.7 ± 6.7	72.2 ± 1.9 *	35.0 ± 0.8 *
7HS + P	196.0 ± 6.6	68.9 ± 3.5 *	35.6 ± 1.4 *

**Table 2:** PCR-primers used in the study.

Gene Description	Primer Sequence
Myh7 (MyHC I(β))	5′-ACAGAGGAAGACAGGAAGAACCTAC-3′ 5′-GGGCTTCACAGGCATCCTTAG-3′
Myh2 (MyHC IIa)	5′-TATCCTCAGGCTTCAAGATTTG-3′ 5′-TAAATAGAATCACATGGGGACA-3′
Myh4 (MyHC IIb)	5′-CTGAGGAACAATCCAACGTC-3′ 5′-TTGTGTGATTTCTTCTGTCACCT-3′
Myh1 (MyHC IId/x)	5′-CGCGAGGTTCACACCAAA-3′ 5′-TCCCAAAGTCGTAAGTACAAAATGG-3′
PGC1alpha	5′-GTGCAGCCAAGACTCTGTATGG-3′ 5′-GTCCAGGTCATTCACATCAAGTTC-3′
SOX6	5′-TCAAAGGCGATTTACCAGTGAC-3′ 5′-TTGTTGTGCATTATGGGGTGC-3′
Rcan1 (MCIP1.4)	5′-CCGTTGGCTGGAAACAAG-3′ 5′-GGTCACTCTCACACACGTGG-3′
RPL19	5′- GTACCCTTCCTCTTCCCTATGC-3′ 5′- CAATGCCAACTCTCGTCAACAG-3′
COXI	5′-ATTGGAGGCTTCGGGAACTG-3′ 5′-AGATAGAAGACACCCCGGCT-3′
COXII	5′-ATTGGAGGCTTCGGGAACTG-3′ 5′-AGATAGAAGACACCCCGGCT-3′
COXIV	5′-TGGGAGTGTTGTGAAGAGTGA-3′ 5′-GCAGTGAAGCCGATGAAGAAC-3′
TFAM	5′-CGCCTGTCAGCCTTATCTGTA-3′ 5′-TGCATCTGGGTGTTTAGCTTA-3′

## Data Availability

The data presented in the study are available upon reasonable request from the corresponding author.
